# Characterization of trimethoprim resistant *E. coli* dihydrofolate reductase mutants by mass spectrometry and inhibition by propargyl-linked antifolates†
†Electronic supplementary information (ESI) available: Supplemental Fig. 1–7. These figures include the sequence of DHFR, typical MS1 spectra of DHFR complexes, SEC elution profiles of DHF, UVPD mass spectra of P21L·NADPH complexes and sequence maps, difference plots of UVPD backbone cleavage propensities for binary and ternary complexes, and cell growth curves. See DOI: 10.1039/c6sc05235e


**DOI:** 10.1039/c6sc05235e

**Published:** 2017-03-28

**Authors:** Michael Cammarata, Ross Thyer, Michael Lombardo, Amy Anderson, Dennis Wright, Andrew Ellington, Jennifer S. Brodbelt

**Affiliations:** a Department of Chemistry , University of Texas , Austin , TX 78712 , USA . Email: jbrodbelt@cm.utexas.edu; b Center for Systems and Synthetic Biology , University of Texas , Austin , TX 78712 , USA; c Department of Pharmaceutical Sciences , University of Connecticut , Storrs , CT 06269 , USA

## Abstract

Pathogenic *Escherichia coli*, one of the primary causes of urinary tract infections, has shown significant resistance to the most popular antibiotic, trimethoprim (TMP), which inhibits dihydrofolate reductase (DHFR). The resistance is modulated by single point mutations of DHFR. The impact of two clinically relevant mutations, P21L and W30R, on the activity of DHFR was evaluated *via* measurement of Michaelis–Menten and inhibitory kinetics, and structural characterization was undertaken by native mass spectrometry with ultraviolet photodissociation (UVPD). Compared to WT-DHFR, both P21L and W30R mutants produced less stable complexes with TMP in the presence of co-factor NADPH as evidenced by the relative abundances of complexes observed in ESI mass spectra. Moreover, based on variations in the fragmentation patterns obtained by UVPD mass spectrometry of binary and ternary DHFR complexes, notable structural changes were localized to the substrate binding pocket for W30R and to the M20 loop region as well as the C-terminal portion containing the essential G–H functional loop for the P21L mutant. The results suggest that the mutations confer resistance through distinctive mechanisms. A novel propargyl-linked antifolate compound 1038 was shown to be a reasonably effective inhibitor of the P21L mutant.

## Abstract 2

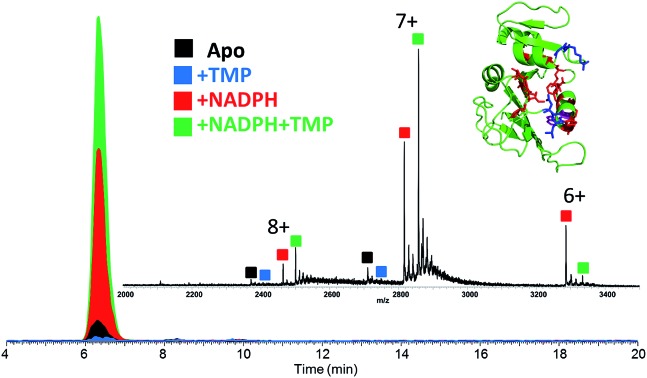
Native mass spectrometry, size exclusion chromatography, and kinetic assays were employed to study trimethoprim resistance in *E. coli* caused by mutations P21L and W30R of dihydrofolate reductase.

## Introduction

Growing concerns about antibiotic-resistant strains of *Escherichia coli*, as well as numerous other pathogenic bacteria, have spurred efforts to expand the pipeline of inhibitors and better understand their interactions with protein targets.^1^–^3^ As one example, dihydrofolate reductase (DHFR) plays a key role in converting dihydrofolate (DHF) into tetrahydrofolate (THF), a process essential for the synthesis of purines and thymidylic acid.^4^ The structures of DHFR in Gram-negative bacteria are distinctive from DHFR in mammalian cells, thus allowing development of inhibitors that are selectively active for bacterial DHFR. Owing to its high affinity for bacterial DHFR, trimethoprim (TMP) has been one of the most widely used antibiotics for the treatment of bladder infections.^1^–^3^,^5^ However, multiple strains of *E. coli* have developed resistance to TMP through chromosomal and point mutations which ultimately modulate the structure of DHFR and allow retention of function even in the presence of previously successful antibiotics.^1^–^3^,^6^–^8^

The ability to assess protein interactions and conformations with high sensitivity by mass spectrometry has proven to be a powerful new approach in recent years. The spectrum of mass spectrometry strategies ranges from those that utilize covalent labelling,^8^ non-covalent labelling^9^ and crosslinking methods^10^ to decipher solvent-accessible regions of proteins in the absence or presence of other proteins, ligands, or inhibitors. Ion mobility methods correlate experimental collision cross-sections with conformations,^11^ and other approaches use native mass spectrometry to evaluate stoichiometries of protein complexes^12^–^14^ and to associate fragmentation patterns with conformational variations.^15^,^16^ Native mass spectrometry entails electrospraying proteins from solutions containing high concentrations of volatile salts to aid the preservation of non-covalent interactions and native-like conformations of proteins as they are transferred to the gas phase. Native mass spectrometry has been applied to systems ranging from membrane proteins to whole virus capsids, revealing biologically relevant insights that have opened new frontiers in the application of mass spectrometry to the field of structural biology.^17^–^20^ Although the structural resolution obtained from mass spectrometry-based methods rarely rivals that obtained from NMR and X-ray crystallography methods, the speed and low sample consumption of mass spectrometry give it several compelling advantages for the characterization of proteins.^8^,^9^,^12^,^21^–^23^

Coupling native mass spectrometry with ion activation techniques such as electron-based methods (ETD or ECD)^24^,^25^ and ultraviolet photodissociation (UVPD)^15^,^16^,^27^ offers a way to extract additional details about the structures of proteins. These details include insight into ligand binding localization,^16^,^26^–^28^ conformational changes,^16^,^29^–^31^ and protein–protein interfacial regions.^32^,^33^ Mass spectrometry can be combined with other auxiliary methods to gain activity/thermodynamic information beyond just structural insight. For example, recently a method that integrated a size-exclusion separation method with mass spectrometry, termed kinetic size exclusion chromatography (SEC), was developed to determine dissociation constants of protein–ligand complexes.^34^,^35^ Native MS has been combined with SEC or ion exchange chromatography to study complex protein systems such as oligomers of bovine serum albumin (BSA)^36^ and protein conjugates for biotherapeutics.^37^ We build on this prior SEC-MS work in the present study as a means to gain qualitative thermodynamic information in the form of protein–ligand dissociation akin to kinetic *K*_off_ values upon migration of DHFR/ligand/inhibitor complexes through the SEC column.

Top-down UVPD-MS has been shown to be a promising method for evaluating conformational variations in proteins upon ligand binding, and we recently used this strategy for deciphering the conformational changes of DHFR and its inhibition by methotrexate (MTX).^16^ Expanding on this previous study, we now report the use of UVPD-MS to explore TMP-resistant DHFR constructs (P21L and W30R variants) in order to characterize the structural changes attributed to single point mutations and determine how the mutations drive antibiotic-resistance. The mechanism of resistance to TMP by DHFR mutations is not yet clear, whether arising from steric hindrance that prevents tight binding of TMP or by induction of a thermodynamic or kinetic shift favoring protein activity over TMP binding.^7^ This type of insight would be useful in designing new inhibitors for future therapeutic uses. Additionally, we employ size exclusion chromatography (SEC) mass spectrometry to monitor the dissociation of DHFR complexes containing co-factor NADPH and an inhibitor (MTX or TMP), a method that allows comparison of the relative *K*_off_ values of DHFR–inhibitor complexes. Specifically, two TMP-resistant DHFR variants (P21L and W30R) clinically isolated from *E. coli* are the focus of the present study.^5^,^6^,^38^ In addition, a new class of DHFR inhibitors, propargyl-linked antifolates (PLAs), has been shown to be potent against an array of different species of DHFR-containing bacteria, including wild-type *E. coli*.^39^–^41^ In the present study, two PLA-based inhibitors are evaluated against the two TMP-resistant *E. coli* mutants as well as WT-DHFR with an emphasis on probing specific interactions that contribute to the inhibition of DHFR. Through the integration of mass spectrometry and kinetic data, the relationship between structure and function is bridged to give a more complete picture of how the P21L and W30R mutations cause TMP-resistance.

## Experimental

### DHFR purification

The *E. coli* folA gene encoding DHFR including a C-terminal His6-tag (amplified from DH10B genomic DNA) was cloned into pACYCDuet-1 (Novagen). P21L and W30R mutations were introduced by QuikChange PCR. BL21(DE3) cells transformed with pACYC-DHFR were diluted 1/250 in 0.5 L of terrific broth and induced with (0.5 mM) IPTG during mid log phase. Cells were harvested by centrifugation at 8000 × *g* for 10 min and resuspended in 25 mL of wash buffer (50 mM K_2_HPO_4_, 300 mM NaCl and 10% glycerol at pH 8.0) with protease inhibitor cocktail (complete, mini EDTA free, Roche) and lysozyme at 0.5 mg mL^–1^. Following 20 min incubation at 4 °C, cells were lysed by sonication (Model500, Fisher Scientific) and clarified three times by centrifugation at 35 000 × *g* for 30 min. Lysate was filtered through a 0.2 μm membrane and DHFR was recovered by IMAC (immobilized metal ion affinity chromatography) using Ni-NTA resin and gravity flow columns. Eluate was concentrated and dialyzed against 50 mM ammonium acetate (pH 6.5) followed by purification to apparent homogeneity by size exclusion FPLC. The sequence of DHFR is provided in Fig. S1.†

### Size exclusion chromatography-mass spectrometry (SEC-MS)

All size exclusion chromatography experiments were performed using a Dionex LC system interfaced to a Thermo Scientific Instruments Velos dual linear ion trap mass spectrometer (San Jose, CA). The LC effluent was introduced using a HESI source with an applied voltage of 4 kV. A 2.1 mm × 30 cm Zenix-C column with 80 Å pore size and 3 μm particle size was used. An isocratic mobile phase comprised of 150 mM ammonium acetate at pH 6.5 was applied at a flow rate of 80 μL min^–1^. Analytical solutions contained 12 μM protein and 25 μM ligand or inhibitor in 150 mM ammonium acetate at pH 6.5 with 2% DMSO to facilitate the solubilization of inhibitors. For each run 5 μg of protein was injected onto column (20 μL injection volume). MS1 spectra over two *m*/*z* regions: *m*/*z* 1500–4000 for proteins and complexes and a narrow low *m*/*z* region around the small molecule inhibitor of interest were collected in an alternating fashion. Experiments were performed in triplicate. Peak areas of the detected protein complexes were tabulated to calculate the proportion of each eluting from the column. The novel propargyl-linked antifolates proved to be very hydrophobic, leading to strong adsorption on the SEC column and preventing monitoring of their elution profiles. This problem might be mitigated by addition of an organic solvent modified at the risk of protein denaturation.

### Native mass spectrometry and UVPD

Solutions (7 μM protein concentration) were loaded into pulled tip silica emitters coated with Au/Pd and sprayed using an applied voltage of 1.9–2.1 kV. All solutions were analyzed using a Thermo Scientific Instruments Orbitrap Elite mass spectrometer (San Jose, CA) outfitted with a 193 nm excimer laser as described earlier.^42^ The buffer contained 150 mM ammonium acetate at pH 6.5 with 2% DMSO. Inhibitors were added to a 2× molar ratio relative to the protein, and NADPH was added to a 5× molar ratio. The 7+ charge state was selected for UVPD fragmentation for each protein or protein complex. Spectra were collected at 120 K resolution at *m*/*z* 400 with an AGC of 1 × 10^5^. For UVPD, proteins were activated using a single 2.5 mJ pulse from an unfocused excimer laser. 200 scans were averaged for each spectrum. Experiments were collected and analyzed in triplicate. Thermo Xtract with a S/N of 2 was used to deconvolute each spectrum. An in-house constructed web-application was used to analyze the resulting deconvoluted data against DHFR sequences with a 10 ppm mass error tolerance. Data was further analyzed as described in ref. 16.

### Growth curve experiments

MG16550 *E. coli* cells were grown and diluted to 0.1 absorbance units at OD of 600 nm. Cells were then incubated at 37 °C with 1 μg mL^–1^ or 10 μg mL^–1^ concentrations of trimethoprim, methotrexate and the two novel PLAs. UV absorbance measurements were recorded at 600 nm for 24 hours every 5 minutes in triplicate.

### Enzyme kinetic and inhibition assays

Enzyme activity assays were performed by monitoring the rate of NADPH oxidation by DHFR *via* absorbance at 340 nm at room temperature (∼25 °C) in buffer containing 20 mM TES (*N*-[tris(hydroxymethyl)methyl]-2-aminoethanesulfonic acid, pH 7.0), 50 mM KCl, 0.5 mM EDTA, 10 mM β-mercaptoethanol, and 1 mg mL^–1^ BSA using 100 μM NADPH and 1.8 μg mL^–1^ of DHFR. Enzyme was mixed and incubated with NADPH for 5 minutes prior to initiation of the enzymatic reaction by the addition of 100 μM DHF in 50 mM TES, pH 7.0. Enzyme kinetic parameters and inhibition data were determined following a standard method that has been previously reported.^40^ For enzymatic inhibition assays, inhibitor in DMSO was added to the enzyme–NADPH mix and allowed to incubate for 5 minutes before initiating the reaction. The inhibitor concentration and volume were based on the conditions that result in a 50% reduction in enzyme activity. Enzyme inhibition was measured in triplicate and the average IC_50_ is reported with standard deviations. Enzyme kinetics were determined by nonlinear regression analysis (GraphPad Prism) of data generated by enzyme activity assays using 12.5–100 μM DHF and 100 μM NADPH to determine the *K*_m_ and *V*_max_ for DHF or 12.5–100 μM NADPH with 100 μM DHF to determine *K*_m_ and *V*_max_ for NADPH.

## Discussion

Our strategy involved integration of several complementary approaches to evaluate the impact of single point mutations (P21L and W30R) on the structure and function of DHFR. Structural characterization was undertaken using a newly emerging method, UVPD-MS, which has proven to be a sensitive means to probe conformational variations based on changes in the fragmentation patterns of proteins and non-covalent protein–ligand complexes.^16^,^17^,^27^ Moreover, the use of size exclusion chromatography allows a reproducible, high throughput manner to introduce the protein–ligand complexes into the mass spectrometer, to compare the relative stabilities of the complexes, and to monitor relative *K*_off_ trends. Michaelis–Menten kinetic and inhibitory kinetic measurements provide a more classical way to evaluate activities of the DHFR variants with respect to the impact of the single mutations.

**Fig. 1:**
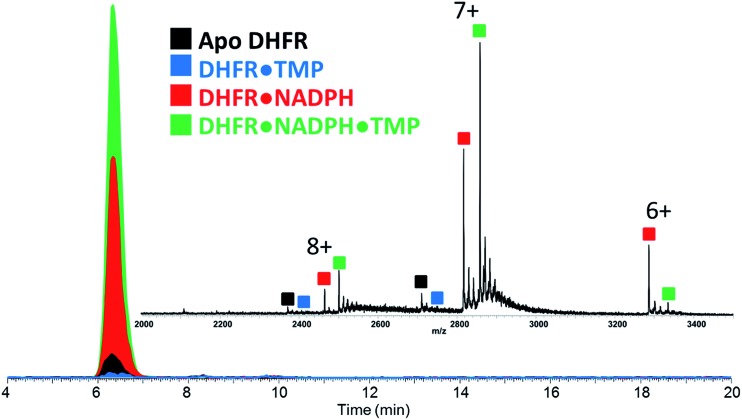
XICs of the protein complexes observed upon size-exclusion chromatography/native MS of a solution containing WT-DHFR, NADPH, and TMP (1 : 5 : 2 molar ratio with a protein concentration of 12 μM). Approximately 5 μg of protein was injected. The inset shows the averaged mass spectrum across the entire SEC peak.

**Fig. 2:**
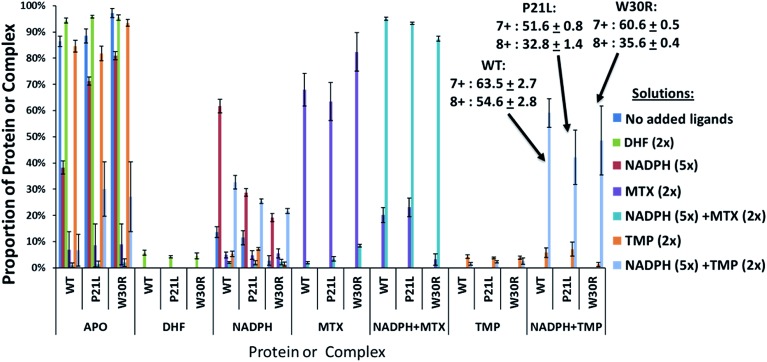
Distribution of complexes detected by native MS during SEC elution for each solution containing WT-DHFR, P21L, or W30R and various added ligands (NADPH, DHF, MTX, and TMP). The concentration of the protein in each solution was 12 μM. The concentration of each ligand was 25 μM. The abundances of the 7+ and 8+ species were summed. Selected percentages and standard deviations are listed for the ternary DHFR·NADPH·TMP complexes (7+ and 8+).

Upon native MS, binary complexes containing co-factor NADPH and DHFR and ternary complexes containing trimethoprim (TMP), NADPH and DHFR were produced for each variant of DHFR (WT, P21L and W30R) (Fig. S2†). Additionally, the MS1 spectra showed that the mutation from tryptophan to arginine for variant W30R did not significantly change the resulting charge states, and the 7+ complexes were favored for all three variants. To shed light upon how the P21L and W30R single-point mutations contribute to TMP resistance, size exclusion chromatography (SEC) coupled to native mass spectrometry was employed to monitor dissociation of the DHFR complexes as a means to evaluate relative *K*_off_ values (where *K*_off_ refers to a kinetic value that describes the rate of dissociation of the protein/ligand complex).^34^,^35^ Solutions containing various ratios and combinations of each DHFR variant (WT or P21L or W30R) with NADPH, DHR, MTX, and TMP were incubated, injected onto the SEC column, and the products were monitored by native MS. Fig. 1 displays extracted ion chromatograms (XIC) for the resulting protein products for one representative solution containing DHFR, NADPH, and TMP in a 1 : 5 : 2 molar ratio. Binary complexes (DHFR·TMP) and (DHFR·NADPH) and ternary complexes (DHFR·NADPH·TMP) as well as the ligand-free protein (apo DHFR) are observed, all in the low 6+, 7+, and 8+ charge states that are characteristic of native MS conditions. Unbound ligands are detected in the low mass range, not shown in Fig. 1. The proportions of each species were determined from the SEC peak areas. The results for each of seven different solutions containing one of the three DHFR constructs and either no ligands, 2× DHF, 5× NADPH, 2× MTX, 5× NADPH + 2× MTX, 2× TMP, or 5× NADPH + 2× TMP are summarized in the histograms in Fig. 2. The three DHFR protein demonstrate striking differences in binding of each of the four ligands (DHF, NADPH, MTX, TMP) as exhibited by the trends in abundances of the binary protein·ligand complexes. SEC-MS analysis of the incubates containing each protein construct and TMP revealed that binary (protein·TMP) complexes were not detected above the baseline noise level for any of the constructs, thus indicating that the binary complexes, if formed, do not survive SEC separation and ESI (Fig. 2). For solutions containing each DHFR protein with substrate DHF, the abundances of binary DHFR·DHF complexes were uniformly very low, whereas for solutions containing inhibitor MTX, the abundances of the binary DHFR·MTX complexes were large for all three proteins (WT, P21L, W30R). The most notable variation in the abundances of the binary complexes occurred for solutions containing co-factor NADPH, for which the binary DHFR·NADPH complexes were significantly more abundant than those containing the P21L and W30R variants, suggesting a substantial modulation of the NADPH binding site upon incorporation of either of the two point mutations.

For solutions containing each protein with co-factor NADPH (5×) plus one of the two inhibitors (MTX or TMP, 2×), the formation of ternary complexes was dramatic compared to the binary complexes summarized above (Fig. 2). In particular, the abundances of the ternary complexes (DHFR·NADPH·TMP) were substantial in contrast to the absence of corresponding binary complexes (DHFR·TMP). This interesting outcome suggests that the co-factor NADPH plays an important role in stabilization of the DHFR·TMP interactions. This result contrasts with the behavior observed for each DHFR protein in the presence of MTX. Abundant complexes of the type DHFR·MTX were observed for each solution containing one DHFR construct and MTX even in the absence of NADPH. When NADPH was added to those same solutions, the abundances of the ternary complexes DHFR·NADPH·MTX were somewhat enhanced relative to the binary DHFR·NADPH or DHFR·MTX complexes, but the increase was modest compared to the striking effect noted for the TMP complexes. In sum, the presence of NADPH was far more critical for production of stable ternary complexes containing TMP than those containing MTX. With respect to variations in formation of ternary complexes for each of the three proteins, W30R produced slightly lower abundances of ternary DHFR·NADPH·MTX complexes (*i.e.* weakest binding) than WT-DHFR or P21L.

**Fig. 3:**
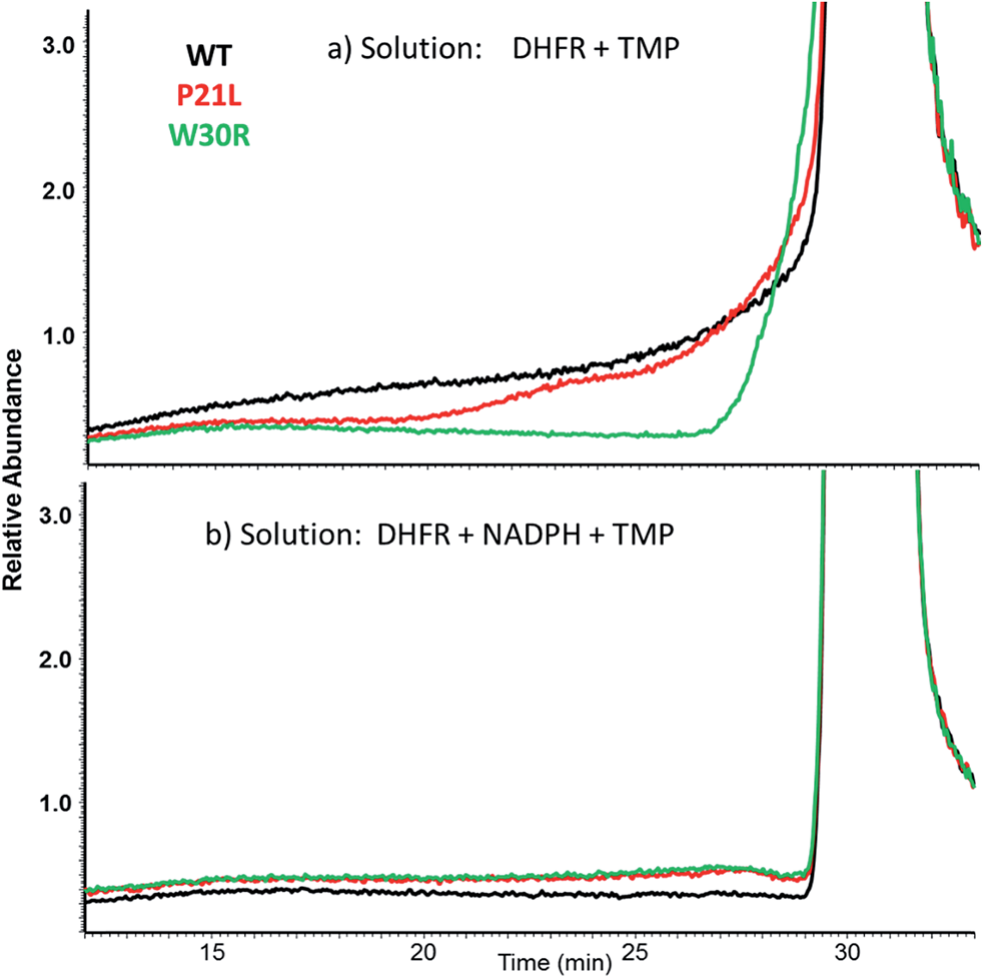
XIC comparisons of SEC-MS elution profiles for TMP from solutions containing (a) protein + TMP or (b) protein + NADPH + TMP for each of the three DHFR constructs. Around 6 minutes, free TMP is detected with the eluting protein. At 30 minutes, unbound TMP is detected.

**Fig. 4:**
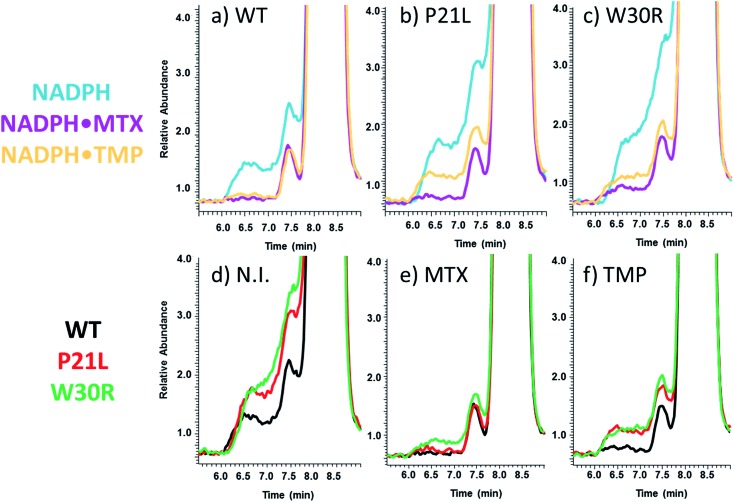
XIC traces (upper series) show elution profiles of NADPH upon SEC-MS analysis of solutions containing protein + NADPH, protein + NADPH + MTX or protein + NADPH + TMP for each of the three DHFR proteins: (a) WT-DHFR, (b) P21L, and (c) W30R. XIC traces (lower series) show elution profiles of NADPH upon SEC-MS analysis of solutions containing WT-DHFR, P21L or W30R with (d) no added inhibitor (N.I.), (e) addition of MTX, and (f) addition of TMP.

SEC-MS was also used to monitor the chromatographic profiles of the unbound ligands, DHF, NADPH, and TMP. Those chromatographic peaks with long fronting tails originate from complexes that dissociate during migration through the column, and the size of the tail correlates with the magnitude of the *K*_off_ values. For example, the elution profiles of TMP show distinctive front shoulders for the incubates containing WT or P21L (but not W30R), indicative of greater *K*_off_ values for these binary DHFR·TMP complexes (Fig. 3a). The fronting is virtually eliminated for solutions containing each protein with NADPH and TMP (Fig. 3b). The same trend of differing *K*_off_ behavior is witnessed for the substrate DHF as well (Fig. S3†). Fig. 4 shows a comparison of the elution profiles for NADPH (based on detection of *m*/*z* 744) for the solutions containing each DHFR protein and NADPH, or each protein and both NADPH and MTX, or each protein and both NADPH and TMP. Fronting (6.0–7.5 minutes) occurs for NADPH released from each of the three protein constructs. There are significant differences in the release of NADPH in the presence or absence of the two inhibitors (MTX *versus* TMP) (Fig. 4a–c) and among the three protein constructs (Fig. 4d–f). The release of NADPH from the binary DHFR·NADPH is lower for WT-DHFR relative to the two mutants. For all three DHFR constructs, addition of either MTX or TMP significantly decreases the release of NADPH from the ternary complexes (*i.e.* lower front prior to the main NADPH peak at 8.0 minutes). These observations provide evidence that these single point mutations exert allosteric effects on the co-factor binding in addition to modulating the binding of each inhibitor.

With respect to examination of changes in the conformation of the protein as a function of ligand binding, UVPD-MS was used to decipher structural differences in the complexes based on variation in the extent of fragmentation along the backbone of the protein (with or without bound ligands). The presumed conformational changes of WT-DHFR upon ligand binding have been examined previously by UVPD-MS, and the detected variations in UVPD fragmentation efficiencies of apo WT-DHFR relative to complexes containing NADPH or methotrexate were found to be consistent with changes in their crystal structures.^16^ In essence, the UVPD fragmentation propensities reflect the efficiencies of backbone cleavages along the protein. Increases of backbone cleavages are presumed to reflect regions of the protein that become more flexible or possibly have enhanced photoabsorption cross-sections owing to variations in secondary structure or other factors, whereas decreases of backbone cleavages are indicative of regions that are more stabilized owing to conformational changes, variations in intramolecular interactions, or other factors.^16^,^17^ An example of a typical UVPD mass spectrum, the sequence coverage map constructed from the UVPD mass spectrum, and the relative extent of backbone cleavage at each position are shown for P21L protein in Fig. S4.† The total protein sequence coverage obtained from the UVPD mass spectrum is 51% for the P21L·NADPH (7+) complex, thus confirming the ability of UVPD to provide extensive information about backbone cleavages throughout the protein. Moreover, large variations in the propensities for cleavages at various backbone positions in Fig. S4† demonstrate the sensitivity of UVPD to structural factors.

**Fig. 5:**
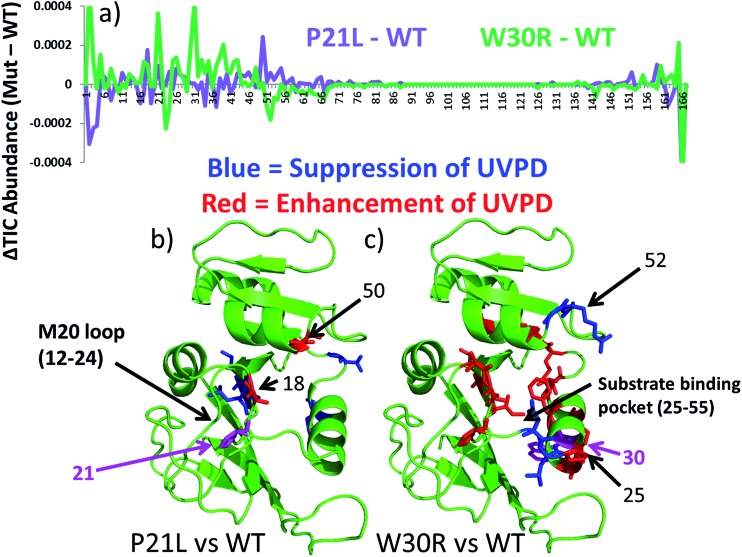
(a) Difference plots of UVPD backbone cleavage propensities for apo P21L (in purple) and apo W30R (in green) relative to apo WT-DHFR. Large differences in UVPD (those with >0.0001 ΔTIC abundance) are highlighted on the DHFR crystal structure (1RX3) for (b) P21L (b) and (c) W30R. Those residues highlighted as blue sticks show suppression of UVPD for the mutant construct relative to WT-DHFR, and those residues highlighted as red sticks show enhancement of UVPD for the mutant construct relative to WT-DHFR. The mutated residue is colored hot pink.

The UVPD mass spectra for matched pairs of protein complexes (*e.g.* WT·MTX *versus* P21L·MTX) are used to create difference plots which allow facile comparison of the changes in backbone cleavage propensities between proteins or their complexes. An example is shown in Fig. 5a for the three apo-proteins, in which the variations in UVPD fragmentation yields (propensities) are shown across the backbone from the N-terminus to the C-terminus for each of the P21L and W30R constructs relative to WT DHFR (*e.g.* comparison of WT to P21L and WT to W30R). The difference plots in Fig. 5a diverge considerably for the two mutant constructs, and the most significant variations are color-coded on the protein structures in Fig. 5b and c. Increases in the propensities of backbone cleavages of the P21L or W30R complexes relative to WT DHFR upon UVPD are highlighted in red, and decreases in the propensities of backbone cleavages are highlighted in blue.

There are relatively modest changes in UVPD fragmentation for the apo P21L mutant relative to apo WT-DHFR (purple trace); however the few changes that are observed are located proximal to the M20 loop (residues 9–25) or in the loop regions surrounding the substrate binding pocket. More substantial variations in UVPD fragmentation covering broader stretches of the backbone occur for the W30R mutant relative to WT-DHFR (green trace), particularly shifts in backbone cleavage propensities in the regions of the substrate/inhibitor binding pocket (residues 25–55) as well as the M20 loop (residues 9–25). Residue 21 of DHFR does not play a major role in the core alpha helix/beta sheet composition of DHFR but rather occupies a peripheral position. This suggests that the P21L point mutation is less likely to cause a significant conformational re-organization of DHFR and instead the mechanism of TMP-resistance may originate from a kinetic or thermodynamic modulation in the uptake of TMP. In contrast, residue 30 is a key amino acid in the core structure of DHFR, and the W30R mutation replaces a hydrophobic amino acid with a more compact hydrophilic residue. W30 is additionally responsible for coordinating a water molecule in the core of the protein.^43^ Upon mutation to arginine the water coordination is less favorable which is also detrimental to the binding of TMP in comparison to that of a P21L or WT construct. The W30R mutation is anticipated to directly modulate TMP binding.^7^

**Fig. 6:**
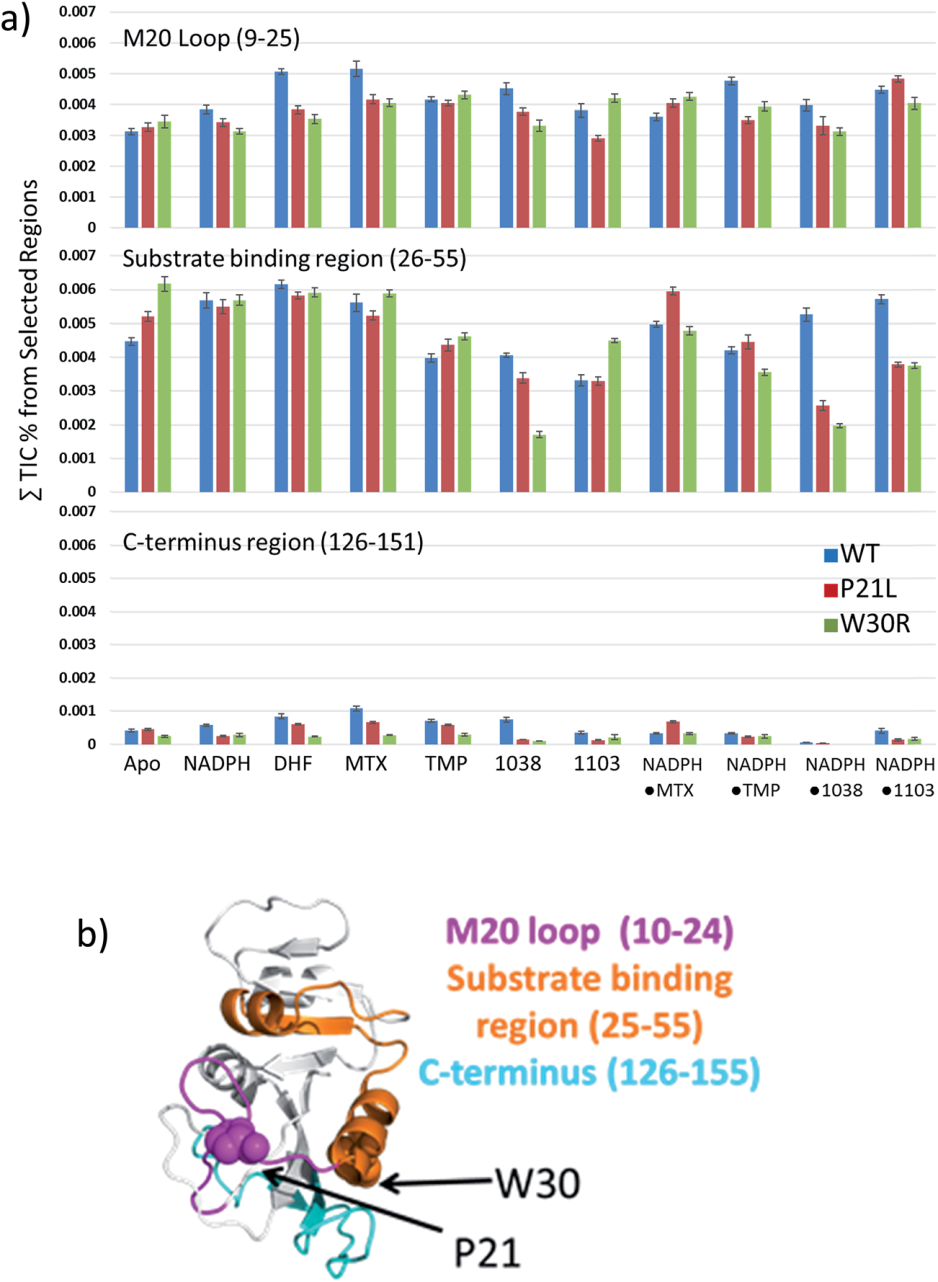
(a) Comparison of differences in UVPD fragmentation efficiencies between each combination of binary and ternary complexes for three structural regions of interest. The structural regions are the M20 loop (residues 9–25), substrate binding region (residues 26–55) and the C-terminus (residues 126–155). The species evaluated by UVPD included each ligand-free protein (apo), binary complexes DHFR·NADPH, DHFR·DHF, DHFR·MTX, DHFR·TMP, DHFR·1038, DHFR·1103, and ternary complexes DHFR·NADPH·MTX, DHFR·NADPH·TMP, DHFR·NADPH·1038, DHFR·NADPH·1103 for each of the three constructs. (b) The three structural regions are highlighted on the DHFR crystal structure (1RX3) with specific residues P21 and W30 shown as space-filled moieties.

Following the use of UVPD-MS to examine the variations in backbone cleavage propensities of the ligand-free (apo) proteins, next binary and ternary protein complexes were characterized. For this phase of the study, complexes containing two propargyl-linked antifolate (PLA) inhibitors (1038 and 1103, structures shown in Fig. S5†) were also included to extend the strategy from well-characterized inhibitors to newly emerging candidates. Each of the binary complexes (DHFR·NADPH, DHFR·DHF, DHFR·MTX, DHFR·TMP, DHFR·1038, DHFR·1103) and ternary complexes (DHFR·NADPH·MTX, DHFR·NADPH·TMP, DHFR·NADPH·1038, DHFR·NADPH·1103) for each DHFR construct (WT, P21L, W30R) were subjected to UVPD-MS to allow examination of the variations in fragmentation patterns (*e.g.* backbone cleavage propensities as a function of ligands and proteins). Examples of the difference plots for UVPD backbone cleavage propensities are shown for binary complexes in Fig. S6† and for ternary complexes in Fig. S7.† The backbone cleavage propensities of the protein backbone were summed for three key regions of the protein (M20 loop comprised of residues 9–25 which contains the first site of mutation (P21), substrate binding region consisting of residues 26–55 which contains the second site of mutation (W30), and G–H loop containing residues 126–151 which is a region essential for protein activity), then plotted as histograms for WT, P21L, and W30R in Fig. 6. UVPD yielded low or no fragmentation of the backbone for one other essential loop (F–G loop, spanning residues 115–130 in the middle of the protein)^44^ and thus could not be evaluated. The comparative histograms for the binary or ternary complexes offer a convenient way to showcase the most significant changes in fragmentation for the large array of binary and ternary complexes.

Conformational changes in the M20 loop modulate the activity of DHFR in *E. coli*. Removing the M20 loop decreases the *K*_cat_ by at least two orders of magnitude, and interestingly in other species the M20 loop is non-functional.^44^–^46^ The M20 loop arranges the co-factor and substrate in proximity for the reduction to occur. The loop goes through an occluded to closed conformation to initiate reduction of DHF to THF and then releases the products upon a third conformational change into the open conformation.^47^ There was a significant decrease in fragmentation of the M20 loop region (residues 9–25) for the binary complexes containing P21L or W30R and the ligands NADPH, DHF, MTX, or 1038 in comparison to the same binary complexes containing the WT protein. A similar decrease in fragmentation of the M20 loop region also occurred for ternary complexes P21L·NADPH·TMP, W30R·NADPH·TMP, P21L·NADPH·1038, W30R·NADPH·1038 compared to the corresponding WT ternary complexes. Suppression of fragmentation upon UVPD is suggestive of stabilization of the protein structure *via* enhancement of non-covalent interactions. Conversely there was a modest increase in fragmentation of the M20 loop region for the ternary P21L·NADPH·MTX and W30R·NADPH·MTX complexes relative to WT·NADPH·MTX. Interestingly, there was a moderate decrease in fragmentation of the M20 loop region for P21L·1103 relative to WT·1103, whereas in contrast there was a moderate increase in fragmentation for the corresponding W30R·1103 complex, suggesting a difference in the way that the two mutations modulate the interaction of DHFR with 1103.

Similar comparisons of fragmentation are possible for the substrate binding pocket region (residues 26–55). In this region the backbone cleavage propensity increased for each of the two mutant constructs relative to WT-DHFR for the apo proteins, suggestive of a less structured, more flexible binding pocket for the two mutant constructs. However, for the various binary and ternary complexes there was notable suppression of fragmentation of the substrate binding pocket for P21L·1038, W30R·1038, P21L·NADPH·1103, W30R·NADPH·1103, P21L·NADPH·1038, and W30R·NADPH·1038 relative to the corresponding WT complexes. This suppression of fragmentation in the substrate binding pocket for P21L and W30R upon complexation with 1038 or 1103 is particularly notable considering that fragmentation of this same region was somewhat enhanced for each apo-protein (P21L and W30R) relative to WT-DHFR, suggesting slight changes in flexibility or accessibility as a result of those mutations. The suppression of fragmentation in the substrate binding pocket for the P21L and W30R mutants upon complexation of 1038 or 1103 may indicate stabilization of that region *via* formation or enhancement of non-covalent interactions between each protein and the PLA inhibitors.

Although the backbone cleavage propensities in the C-terminal region (126–151) were uniformly low for all three proteins and all complexes, in fact the variations in fragmentation (enhancement or suppression) mirrored the changes observed in the M20 loop region for a number of the complexes, specifically ones containing DHF, MTX, or 1038. This is an interesting point as the M20 loop interacts with the G–H loop (residues 141–150) of the C-terminal region. Fragmentation of the C-terminus region is suppressed for the binary P21L and W30R complexes containing NADPH, DHF, MTX, TMP, 1038, or 1103, as well as the ternary NADPH·TMP and NADPH·1103 relative to the corresponding WT complexes. The sole case for which there was a substantial enhancement of backbone cleavages of the C-terminal region occurred for the ternary P21L·NADPH·MTX complexes relative to the WT·NADPH·MTX complexes. In sum, the histograms in Fig. 6 highlight that single point mutations affect the protein structure as evidenced by that enhancement or suppression of backbone cleavages, thus reflecting formation of or release of stabilizing interactions.

To complement the results derived from the SEC-MS and UVPD-MS methods, classical Michaelis–Menten kinetic experiments were performed (Table 1) as well as inhibitory kinetic testing (Table 2). The activity of each DHFR variant was evaluated *via* UV-Vis absorbance measurements by monitoring the conversion of NADPH to NADP^+^ in the presence of substrate DHF at 340 nm (Table 1). Rates of decreasing absorbance at various concentrations of DHF and NADPH were used to calculate *K*_m_ values for DHF and NADPH, respectively. Both variants (W30R and P21L) retained activity for conversion of DHF to THF. As summarized in Table 1, both the P21L and W30R constructs displayed more efficient interactions with DHF relative to WT DHFR based on their smaller *K*_m_ values. W30R showed an eight-fold stronger interaction with NADPH compared to WT-DHFR, whereas P21L displayed significantly weaker interactions for NADPH based on its higher *K*_m_ value. However, this latter factor did not seem to perturb the catalytic constant (*K*_cat_) of the P21L protein which displays a turnover rate similar to that of the WT protein. The W30R construct exhibits an eleven-fold decrease in activity (*K*_cat_) relative to WT DHFR, presumably in part due to the stronger interaction with the co-factor (NADPH). Comparison of the efficiency of the enzyme (*K*_cat_/*K*_m_) for the substrate DHF indicated that the P21L construct was nearly twice as efficient (15.3 (μM s)^–1^) in comparison to WT DHFR. In contrast, the W30R was a third as efficient (2.12 (μM s)^–1^) in comparison to WT DHFR, thus signifying less kinetic fitness for production of tetrahydrofolate (THF). Upon measuring the efficiency for co-factor NADPH, WT DHFR is the most efficient, whereas the P21L and W30R variants show comparable efficiencies with moderately lower values than obtained for WT DHFR. Overall, these results suggest that the DHF binding and release is more greatly affected by the TMP-resistant mutations than are interactions with NADPH.

**Table 1:** Enzyme characterization

DHFR	*K*_m_ [DHF] (μM)	*K*_m_ [NADPH] (μM)	*K*_cat_ (s^–1^)	*K*_cat_/*K*_m_ [DHF] ((μM s)^–1^)	*K*_cat_/*K*_m_ [NADPH] ((μM s)^–1^)
WT	0.81 ± 0.31	4.65 ± 0.56	15.3 ± 0.2	18.8 ± 7.3	3.29 ± 0.40
P21L	2.39 ± 0.26	14.9 ± 2.4	24.2 ± 0.4	10.1 ± 1.1	1.62 ± 0.26
W30R	2.1 ± 1.2	59.3 ± 0.2	1.36 ± 0.04	0.65 ± 0.37	0.0229 ± 0.0006

**Table 2:** Inhibitory kinetic parameters

Compound	DHFR WT		DHFR P21L		DHFR W30R	
	IC_50_ (nM)	*K*_i_ (nM)	IC_50_ (nM)	*K*_i_ (nM)	IC_50_ (nM)	*K*_i_ (nM)
TMP	20.4 ± 2.3	0.165 ± 0.057	220 ± 19	5.14 ± 0.72	478 ± 26	9.7 ± 5.4
MTX	17.5 ± 0.9	0.141 ± 0.054	15.1 ± 0.5	0.352 ± 0.040	11.2 ± 0.3	0.29 ± 0.13
1038	31.0 ± 7.1	0.25 ± 0.11	38.2 ± 3.1	0.89 ± 0.12	130 ± 11	2.6 ± 1.5
1103	54.7 ± 1.4	0.44 ± 0.39	117 ± 9	2.73 ± 0.36	296 ± 3	6.0 ± 3.4

While direct comparisons between *K*_m_ values from the Michaelis–Menten experiments and the qualitative *K*_off_ values obtained from the SEC-MS measurements cannot be made, they provide complementary kinetic and thermodynamic information. *K*_m_ is indicative of protein–ligand binding (DHF and NADPH), enzymatic activity, and release of products (THF and NADP^+^) over the course of the reaction. Relative *K*_off_ values reveal information about the rate of release of the ligands (DHF and NADPH) or inhibitor (TMP) and stabilities of the protein complexes. These are two perspectives when describing the enzymatic landscape. First, the SEC-MS results revealed that binary protein·NADPH complexes were most stable for WT-DHFR followed by the P21L and W30R constructs. Among the three proteins, the *K*_off_ value for TMP was similar for WT-DHFR and P21L in the binary complexes. However, for the corresponding ternary DHFR·NADPH·TMP complexes the *K*_off_ values for TMP were virtually identical for all three protein constructs. The *K*_off_ values for co-factor NADPH were significantly greater for the ternary DHFR·NADPH·TMP and DHFR·NADPH·MTX complexes relative to the binary DHFR·NADPH complexes (Fig. 4). Interestingly, both P21L and W30R display higher affinities (lower *K*_m_ values) than WT-DHFR for DHF which may be reflective of a lower energy dissociation pathway for substrate binding and subsequent product release. However, in the case of W30R the *K*_cat_ is diminished by an order of magnitude suggesting a significantly slower product release, an outcome that originates from the substantially lower *K*_m_ [NADPH] for W30R. The P21L construct has the lowest affinity (*K*_m_) for NADPH, which is consistent with the location of the point mutation on the M20 loop which is responsible for NADPH binding.

Complementarity between the thermodynamic *K*_off_ and kinetic parameters is revealed by comparing the IC_50_ and *K*_i_ values of the positive control inhibitor TMP. Trimethoprim (TMP) is a well-known inhibitor of DHFR with a nanomolar binding constant (Table 2). IC_50_ values for the two DHFR variants, W30R and P21L, exhibited a 23-fold and 11-fold increase, respectively, in the inhibitory concentration compared to WT DHFR, thus confirming significant resistance to TMP. In addition, the fact that the *K*_i_ value for TMP is greater for W30R than P21L indicates that the W30R construct is more susceptible to exchanging the inhibitor for the substrate. In short, the W30R construct exhibits lower affinity for TMP than do the WT or the P21L proteins. The outcomes from the inhibitory kinetics of TMP align with the structural insight garnered from the UVPD-MS trends. These trends indicated that there was a larger conformational change in the binding pocket of W30R in comparison to the WT and P21L proteins. The conformational changes of the substrate binding pocket of W30R may facilitate the greater exchange of the TMP inhibitor for the substrate and account for the doubling of the IC_50_ value for TMP in comparison to the P21L construct. Based on the UVPD fragmentation data, conformational changes were not observed for the substrate binding pocket for P21L in comparison to WT-DHFR. For P21L significant conformational change was witnessed in the M20 loop and C-terminal portion of the ternary complexes, specifically for (DHFR·NAPDH·MTX) and (DHFR·NADPH·1103). The above evidence is suggestive that the two mutations induce resistance through two separate mechanisms. Additionally, the negative control inhibitor (MTX) did not cause a large shift in either the IC_50_ nor the *K*_i_ values for either of the two DHFR variants.

To further parse out differences in mechanisms of resistance (and inhibition) for the two TMP-resistant mutants, the activities of two novel propargyl-linked antifolates (PLAs) were tested against wild type *E. coli* MG1655 cells to determine if either one exhibited positive inhibitory activities on a cellular level (Fig. S5†). The candidate inhibitors, 1038 and 1103, caused a significant decrease in cell growth at 1 μg mL^–1^. The TMP control quenched nearly all cell growth at 1 ug mL^–1^, whereas the negative control MTX showed no inhibition of cell growth. It has been reported that MG1655 cells have efflux pumps which render MTX ineffective *in vivo*.^48^ Additionally, the physiochemical profile of MTX is less favorable from an antibacterial standpoint. Given the relatively high molecular weight and highly polar profile of MTX, it does not lend itself to diffusion across bacterial cell membrane, thus making it ineffective despite the ability to inhibit DHFR. The PLA class of antifolates, however, has been specifically designed to inhibit TMP-resistant enzymes. The inhibition of each of the three DHFR constructs by the two PLAs (1038 and 1103) is summarized in Table 2 along with the comparative results for inhibitors TMP and MTX.

Interestingly, the PLA compounds are able to maintain activity in the mutant enzymes relative to TMP. The UVPD fragmentation results for P21L suggests that TMP resistance is dependent on a change in enzyme kinetics and distinctly different than W30R. This conclusion is supported by the IC_50_ data presented in Table 2. Compound 1103 behaves similarly to TMP which can be anticipated as it more closely resembles TMP in size, molecular weight, and flexibility. Upon evaluation of the effectiveness of inhibition of WT-DHFR relative to the P21L mutant, TMP experiences a 10-fold loss in IC_50_ and 1103 a 2-fold loss compared to 1038 and MTX, both of which are able to maintain potency. Neither TMP nor 1103 engage in same degree of electrostatic and hydrophobic interactions as MTX or 1038 and as a result could be more sensitive to the mutant enzyme's catalytic/conformational changes. In contrast, UVPD fragmentation data for W30R suggests the W30R mutation causes resistance through enthalpic changes and a direct thermodynamic change owing to loss of contacts between the ligand and the enzyme. This conclusion carries through in inhibition values as well. TMP demonstrates a 25-fold decrease in potency for inhibition of W30R relative to WT DHFR, whereas 1103 and 1038 experience less of a penalty with only a 6-fold and 4-fold loss, respectively. It should be noted that the *K*_cat_ of the W30R mutant reported by Watson, *et al.*^7^ is 264 s^–1^ compared to 1.36 s^–1^ reported here. The buffer conditions for the kinetic measurements are not identical for the current *versus* previous study^7^ and a different enzyme monitoring method was used as well (fluorescence *versus* absorbance). However, the enzyme inhibition values correlate well with a functioning enzyme as TMP clearly loses activity. MTX would not be expected to lose potency across these mutants as it so closely mimics the natural substrate, DHF, and relies heavily on strong electrostatic interactions with the enzyme for binding. High-resolution crystal structures of wildtype *E. coli* DHFR bound to MTX^44^ show that W30 is responsible for engaging in a hydrogen bond with the substrate or ligand. The W30R mutation is quite drastic and would be anticipated to dramatically alter this interaction. The enzyme inhibition data suggests that TMP relies most heavily on this interaction and the PLAs less so. Without crystal structures of PLAs, we look to crystallographic evidence of PLAs in complex with *K. pneumoniae* DHFR, which shares 92% sequence identity, to explain why PLAs retain activity relative to TMP.^49^ These compounds make extensive contacts with a hydrophobic pocket consisting of F31, T46, I50 and L54 that is conserved in *E. coli* DHFR. These results further convey the large differences in the modes of action caused by the two point mutations of DHFR and structure-related effects on resistance.

## Conclusions

Based on integration of native MS, UVPD, and SEC data along with conventional Michaelis–Menten and inhibitory kinetic measurements, the structure–function relationships of TMP-resistant DHFR mutants were evaluated. Two different mechanisms of resistance were revealed, one which directly involved modulation of the core structure of the protein (W30R), whereas the other instilled resistance by adjustment of the rigidity of the M20 loop to aid in TMP release (P21L). Overall the variations in the qualitative *K*_off_ trends from the SEC-MS experiments agreed with the trends based on the IC_50_ and *K*_i_ values. Perhaps an even more exciting finding is that compound 1038 which been shown previously to be very potent for inhibition of *E. coli* DHFR is also potent for the P21L TMP-resistant constructs. These findings support the promise of new propargyl-linked antifolates as effective inhibitors of DHFR in pathogenic bacteria.

## Supplementary Material

Supplementary information
