# Supplementary material for: Characterization of trimethoprim resistant E. coli dihydrofolate reductase mutants by mass spectrometry and inhibition by propargyl-linked antifolates
Source: Chem Sci. 2017 Mar 28;8(5):4062–72. doi: 10.1039/c6sc05235e (PMC6020862; doi:10.1039/c6sc05235e)

Electronic Supplementary Material (ESI) for Chemical Science.
This journal is © The Royal Society of Chemistry 2017

Characterization of Trimethoprim Resistant *E. coli* Dihydrofolate Reductase Mutants by Mass Spectrometry and P21L Inhibition by Propargyl-Linked Antifolates

Michael Cammarata[1], Ross Thyer[2], Michael Lombardo[3], Amy Anderson[3], Dennis Wright[3], Andrew Ellington[2], Jennifer S. Brodbelt[1*]

Department of Chemistry[1]

Center for Systems and Synthetic Biology[2]

University of Texas

Austin, TX 78712

Department of Pharmaceutical Sciences[3]

University of Connecticut

Storrs, CT 06269

Correspondence to: jbrodbelt@cm.utexas.edu

Supporting information

Supplemental Figure 1. DHFR sequence with P21 and W30 highlighted, in red and blue, respectively to distinguish the mutated residues. The C-terminal 6XHis tag is included in the sequence.

MISLIAALAVDRVIGMENAMPWNLPADLAWFKRNTLNKPVIMGRHTWESIG
RPLPGRKNIILSSQPGTDDRVTWVKSVDEAIAACGDVPEIMVIGGGRVYEQF
LPKAQKLYLTHIDAEVEGDTHFPDYEPDDWESVFSEFHDADAQNSHSYCFE
ILERRGSHHHHHH

Supplementary Figure 2. ESI mass spectra for solutions containing one of the three constructs of DHFR (WT, P21L, or W30R) with TMP (2X) and NADPH (5X) in 150 mM ammonium acetate and 2% DMSO.

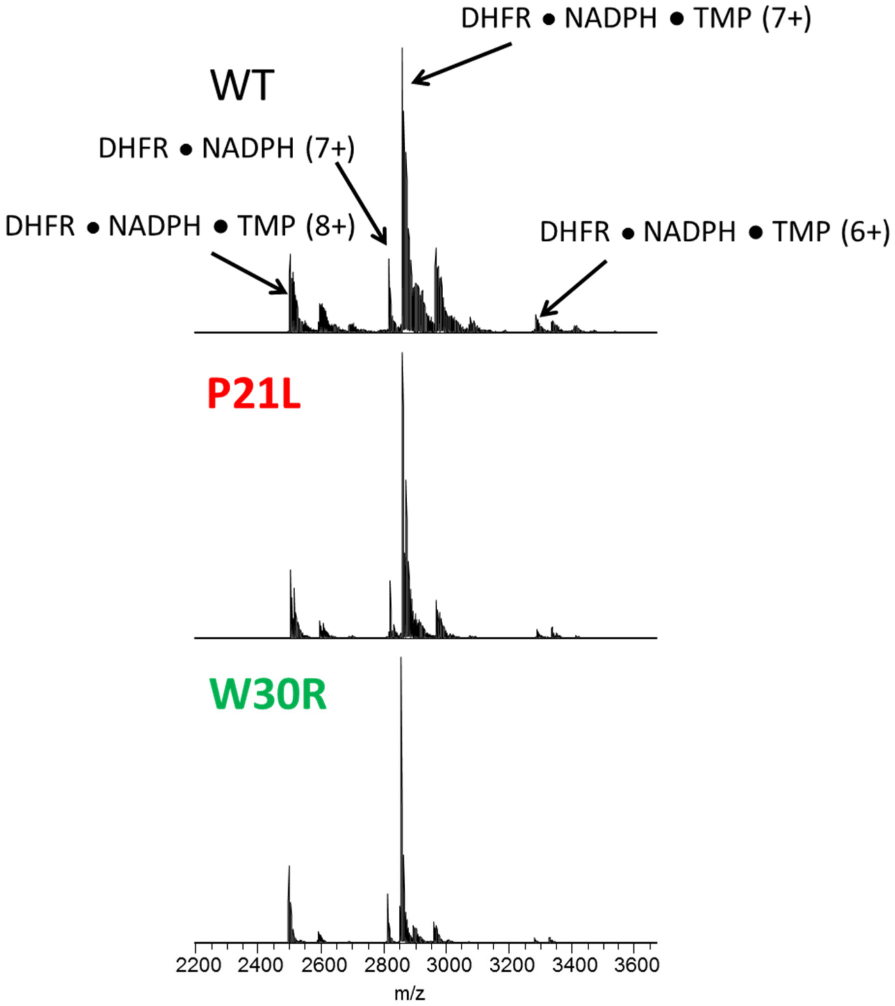

Supplementary Figure 3. Extracted ion chromatograms (XICs) of elution profiles for dihydrofolic acid (DHF) from solutions containing protein + DHF with respect to each DHFR construct (WT, P21L, W30R).

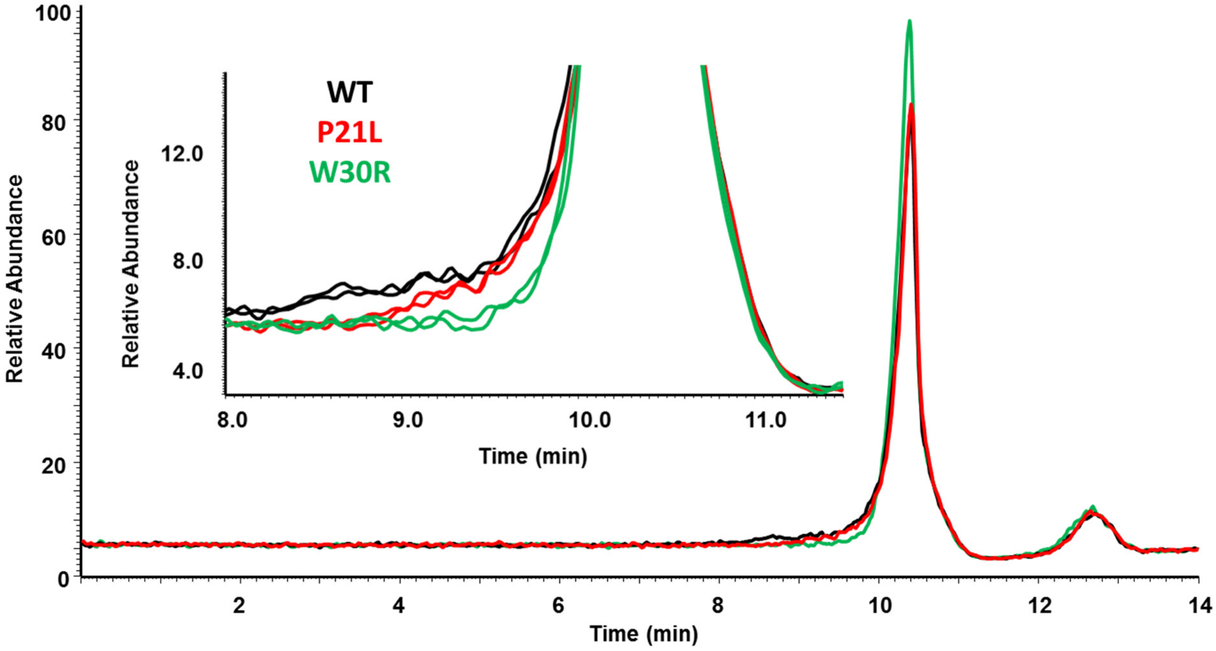

Supplementary Figure 4. a) UVPD mass spectrum of the P21L●NADPH (7+) complex. b) Cleavage map of all apo ions (fragments that do not contain NADPH) and c) cleavage map of all holo ion ions (fragments that contain NADPH (+745.0906 Da)). D) The summed abundances of both apo and holo ions are shown across the backbone of the protein.

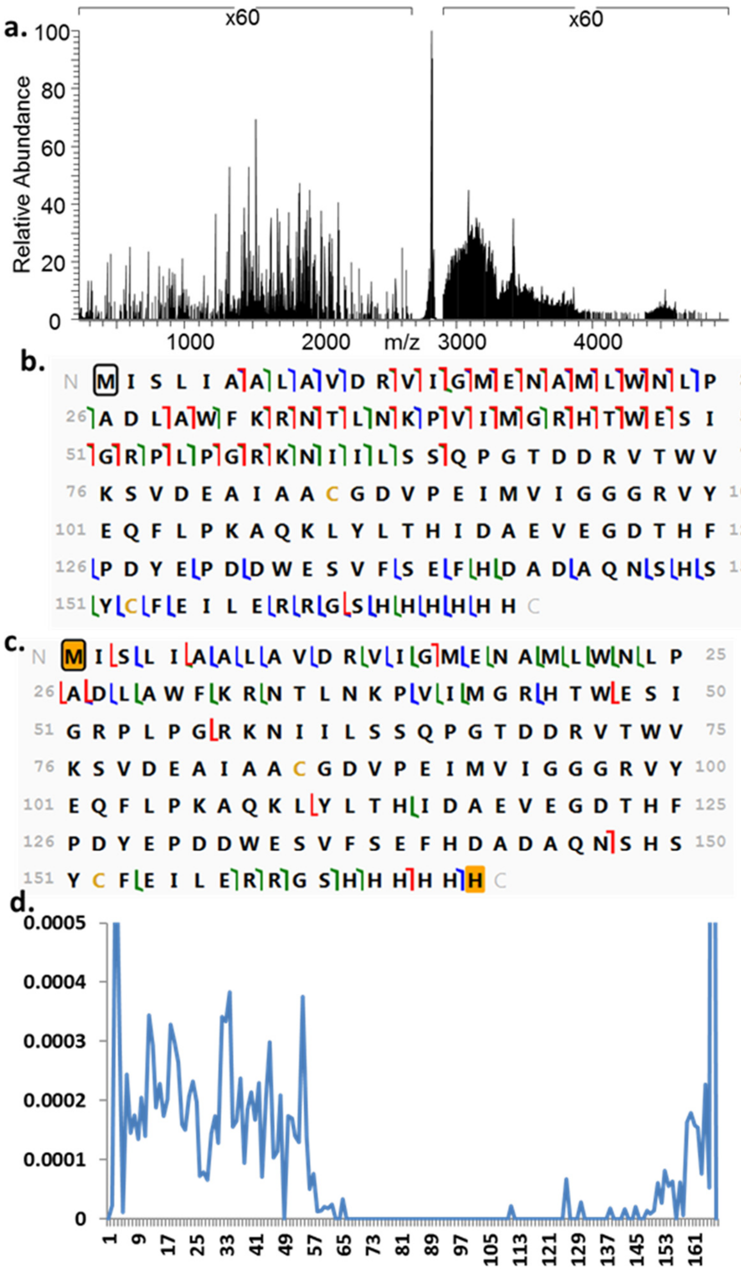

Supplementary Figure 5. Growth curves of *E. coli* MG1655 cells incubated with 1 µg/ml inhibitor (compound 1038, 1103, MTX or TMP) relative to the control (no inhibitor added). The structures and IC_{50} (nM) values for three inhibitors of WT DHFR are shown.

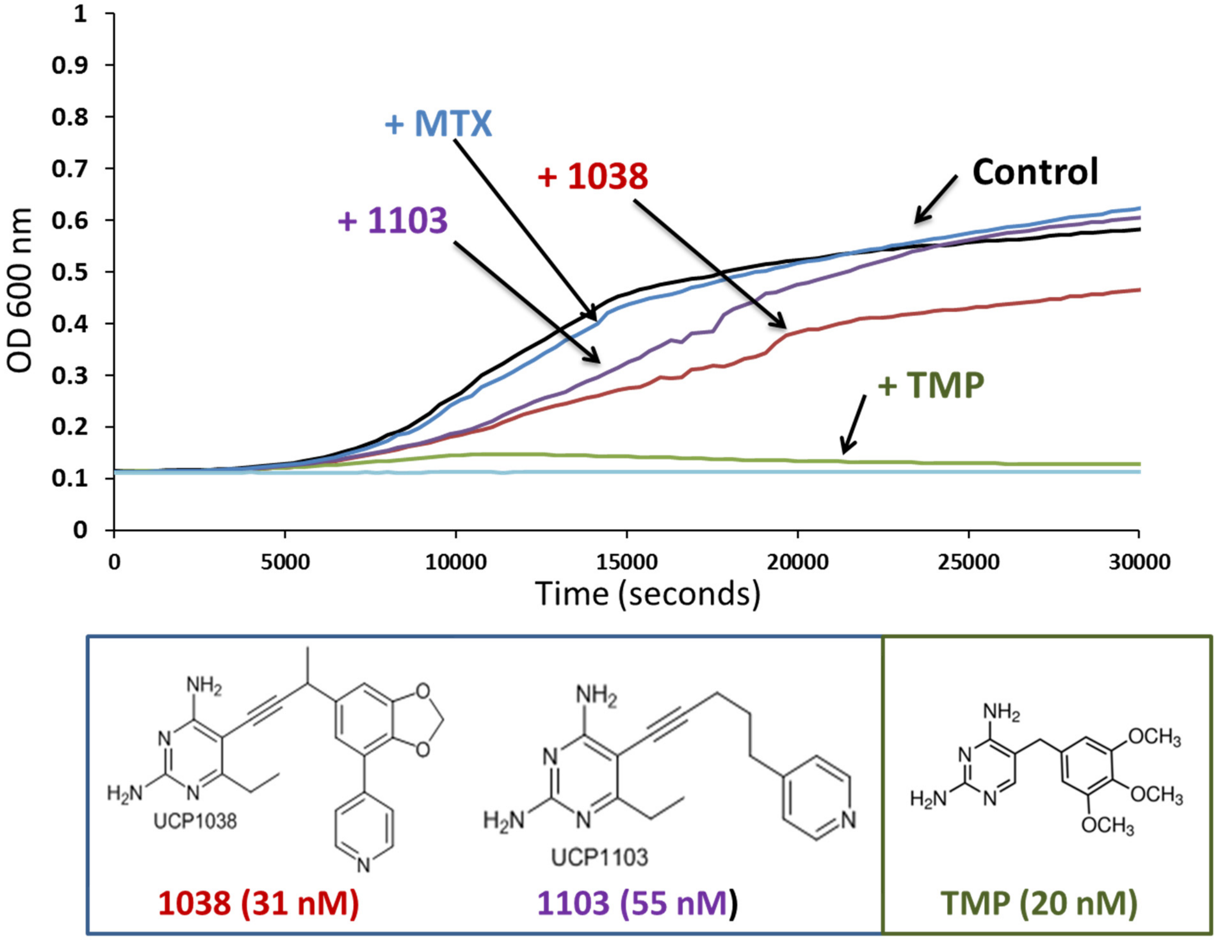

Supplemental Figure 6. Difference plots of UVPD backbone cleavage propensities for binary complexes: P21L●MTX - WT●MTX and W30R●MTX - WT●MTX; P21L●TMP - WT●TMP and W30R●TMP - WT●TMP ; and P21L●1038 - WT●1038 and W30R●1038 - WT●1038.

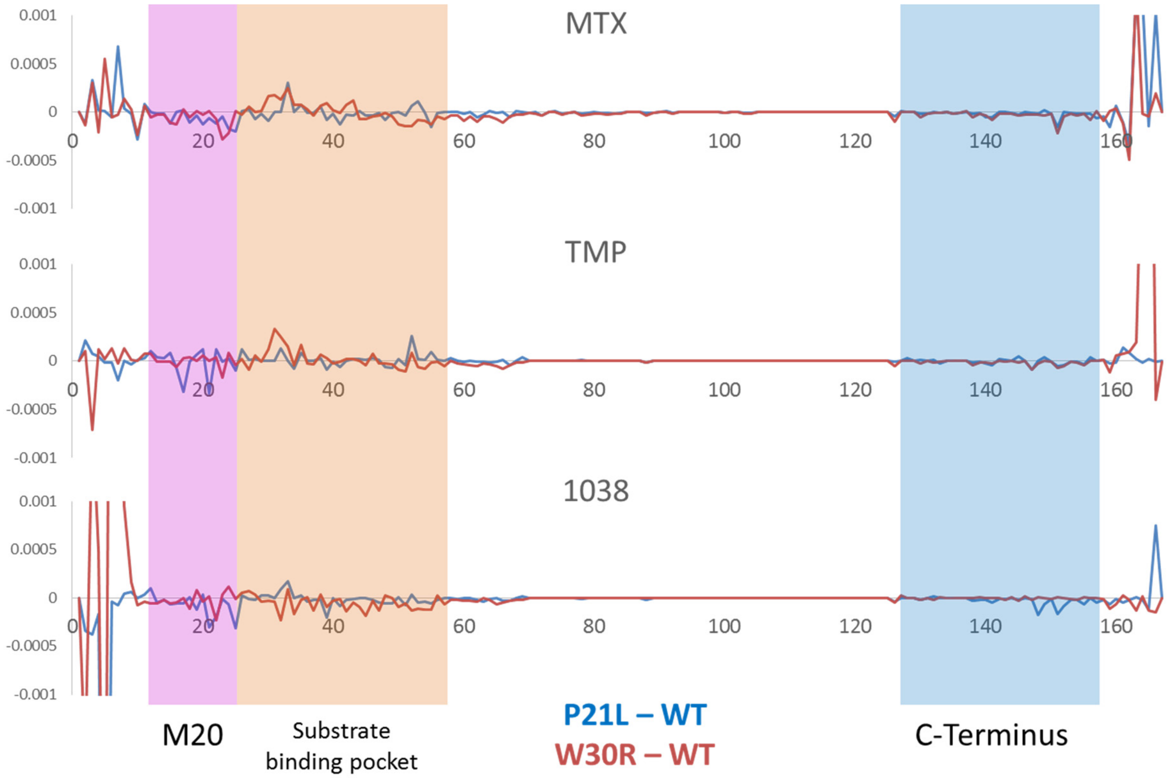

Supplemental Figure 7. Difference plots of UVPD backbone cleavage propensities for P21L●NADPH●MTX - WT●NADPH●MTX and W30R●NADPH●MTX - WT●NADPH●MTX; P21L●NADPH●TMP - WT●NADPH●TMP and W30R●NADPH●TMP - WT●NADPH●TMP; and P21L●NADPH●1038 - WT●NADPH●1038 and W30R●NADPH●1038 - WT●NADPH●1038.

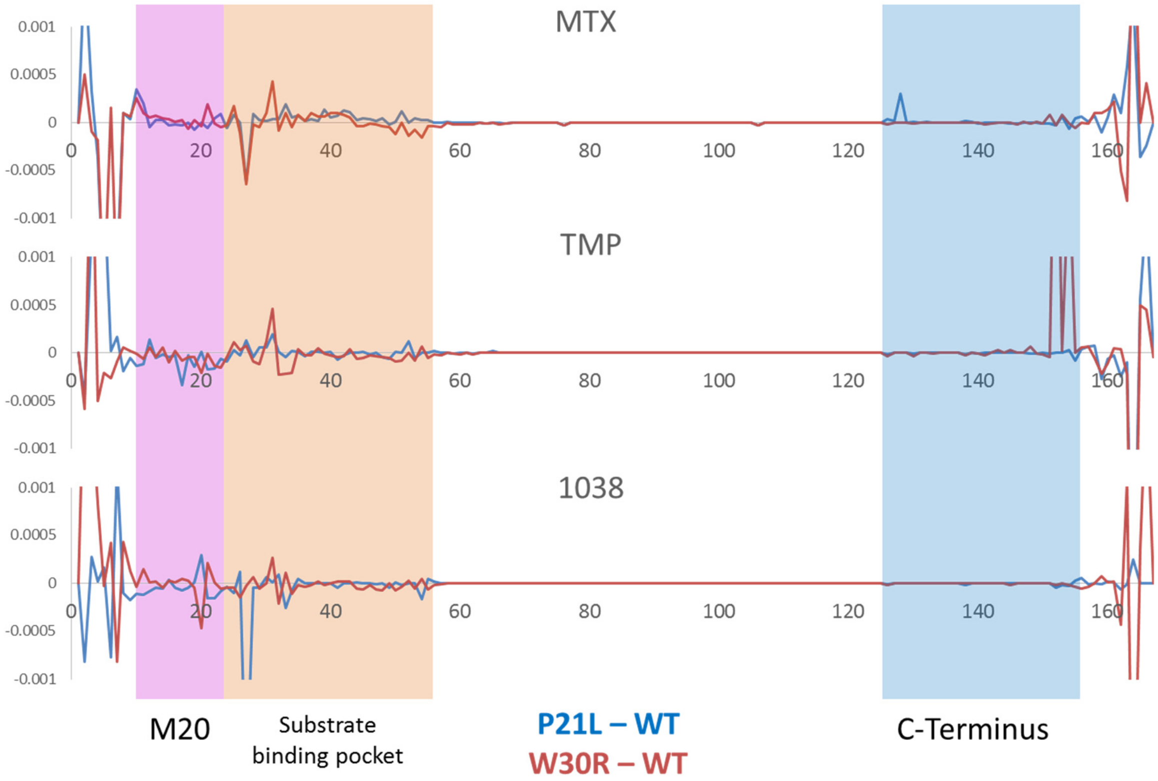

Supplement: Supplementary file 1 [file SC-008-C6SC05235E-s001.pdf]
